# Supplementary material for: A Systematic Review and Meta-Analysis of Anti-Rheumatic Drugs and Pneumococcal Vaccine Immunogenicity in Inflammatory Arthritis
Source: Vaccines (Basel). 2023 Nov 2;11(11):1680. doi: 10.3390/vaccines11111680 (PMC10674424; doi:10.3390/vaccines11111680)
Supplement: Supplementary file 1 [file vaccines-11-01680-s001.zip › vaccines-2637034-supplementary.pdf]

Supplemetary Material

Table S1. Studies included in the metaanalysis.

Author, Year (ref)	Number of subjects (N)	Vaccine Intervention	Outcome	Age, years (mean, SD unless specified)	% Female	SC %	SP %	NOS
Bingham 2010	Rituximab (68) Methotrexate (32)	23 valent	≥2-fold increase or an increase of 1 mcg/ml from pre vaccination level	RTX + MTX 49.7 (9.6) MTX 49.7 (10.5)	RTX + MTX (75) MTX (78)	RTX+MTX (57) MTX (82)	-	8
Bingham 2015	Tocilizumab & Methotrexate (60) Methotrexate (31)	23 Valent	≥2-fold increase or an increase of 1 mcg/ml from pre vaccination level, to ≥6/12 serotypes	TOC + MTX 51.1 (8.9) MTX 51.4 (9.5)	TOC+MTX (75.9) MTX (81.5)	TOC + MTX (60) MTX (70.8)	-	8
Elmer 2022	Methotrexate (11) HC (13)	23 valent	2 fold increase in >6 of the antibody serotypes	Median, IQR MTX 62.9 (58.3-66.7) HC 41.1 (35.9-56.5)	MTX (91) HC (61.5)	MTX (56) HC (90)	-	8
Kaine 2007	TNFi (99) Control (109)	23 valent	SC = >2 fold tire increase in 3/5 antigens. SP = >1.6ul for 3/5 antigens	51.7 (11.66)	79.8%	Placebo (37.4) TNFi (40.4)	Placebo (81.7) TNFi 85.9	8
Kapetanovic 2006	TNFi (62) TNFi + MTX (50) MTX (37) HC (47)	23 valent	≥ 2-fold or higher increase in 6b and 23f serotype	Median, IQR TNFi 53.7 TNF+MTX 52.8 MTX 61.3 HC 30.3	TNFi (76) TNFi + MTX (70) MTX (68) HC (74)	TNFi (50) TNFi + MTX (32) MTX (13.5) HC (38.2)	-	8
Kapetanovic 2011	MTX (85) TNF+MTX (89) TNF (79) Control (85)	23 valent	ARR ≥ 2, 4 to 6-weeks post-vaccination, serotypes 6b and 23f	MTX 61.5 (14) TNFi + MTX 60.1 (10) TNFi 59.8 (14) Control 51.6 (12)	MTX: (78.8) TNFi + MTX (77.5) TNFi (87.3) Control (45.3)	MTX 18 (21.2) TNFi + MTX (15.7) TNFi 29 (36.7) Control 41 (47.7)	-	8
Kapetanovic 2013	RTX ± MTX (55) ABA ± MTX (17) TOC ± MTX (16)	PCV7	ARR ≥ 2, 4-6 weeks post-vaccination, serotypes 6b and 23f	60.2 (2.0)	74			8

Kvitz 2014	Placebo +/- MTX (110) CZP+/- MTX (107)	23 valent	≥ 2-fold increase in ≥ 3 of 6 pneumococcal antigens at 6 weeks, serotypes: 6b, 9v, 14, 18c, 19f, 23f	Placebo 52.7 (11.1) CZP 53.1 (11.8)	Placebo (76.3) CZP (83.6)	Placebo (62.5), CZP (54.5)	-	7
Migita 2015	MTX (55) ABA (21) Control (35)	23 valent	≥2-fold increase in IgG concentrations of 6b or 23f serotypes	Control 70.5 (10.8) MTX 63.8 (11.5) ABA 59.8 (12.0)	Control (65.7) MTX (80) ABA (81)	Fold increase in GMT 6b (95%CI)/23f (95% CI) RA control 2.38 (1.41 - 5.62)/3.36(1.85 to 9.42) MTX 1.75(1.15-3.11)/ 2.00(1.27 to 5.48) ABA 1.41(0.87-3.09)/ 2.45 (1.23- 7.44)	-	8
Visvanathan 2007	IFX 3mg/kg +MTX (20) IFX 6mg/kg + MTX (36) Placebo + MTX (14)	23 valent	≥ 2-fold increase at least 6 vaccine serotypes compared to pre-vaccine levels	Median, IQR IFX 3mg/kg: 52 IFX 6mg/kg 50 Placebo 50	IFX 3mg/kg (65) IFX 6mg/kg (66.7) Placebo (78.6)		-	6

Table S2. Studies not included in the meta-analysis.

Author, Year	Subjects (N)	Vaccine	Outcome	Age, years (mean, SD unless specified)	Female (%)	Vaccine Response (%)	Comments
Alten 2016	ABA + MTX (115) ABA(10)	23 valent	≥2-fold increase in post-vaccination titres to ≥3 of 5 antigens and protective Ab levels of ≥1.6 mcg/mL to ≥3 of 5 antigens.	45.7 (13.8)	85.6	83.9 %	Serotypes measured 9V, 14, 18C, 19F, 23F. No comparator group
Fischer 2015	Corticosteroids (35) Other (181)	23 valent	SC = IGG 0.5ul in 4/6 serotypes at 4-8 weeks	Median, IQR 53.9 (43.3-65.7)	53.2	87%	56 received PPV23, 141 observation arm. Systemic steroids give poor serological response to vaccine.
Garrido 2022	Prednisolone (38), thiopurine (28) calcineurin inhibitor (25) MMF (27)	PPSV13 + 23 valent	seroprotection at 4 months >1.3ug/ml rise in 70% of serotypes	41 (26)	53	TNF (59) Other biologics (45) Il12-23 (43) Rituximab (6) Vedolizumab (70%)	combination therapy reduced seroprotection (44%). Rituximab gives poor humoral response (6%).

	Other (4)<br>DMARD + Other (3)<br>TNF (60)<br>IL12-23 (7)<br>Rituximab (8)<br>Ocrelizumab (11),<br>Tofacitinib (3)<br>Seckukinumab (2)<br>Vedolizumab (10)<br>5-ASA (15)<br>Control (36)					5-ASA (68)<br>DMARD monotherapy (57)<br>DMARD + other (54)	
Lee 2014	5ASA (37)<br>IM (70)<br>TNFi (40)<br>TNFi+IM (50)	23 valent	pre and post IgG response 4 weeks	5ASA 36.0 (19.0-65.0), IM 26.5 (24.0-48.0), TNFi 32.0 (18.0-48.0), TNFi+IM 30.5 (16.0-57.0)	ASA (79)<br>IM (52)<br>TNFi (74)<br>TNFi+IM (64)	Fold increase: GMT ASA 1.373 (1.298-1.447), IM 1.368 (1.332-1.414), TNFi 1.249 (1.86-1.312), TNFi+IM 1.275 (1.222-1.329)	Having both crohns disease and being medicated with a TNF inihibtor hinder the vaccine response.
Mease 2003	MTX(83)<br>Corticosteroids (22)<br>Etanercept (94)	23 valent	2 and 4 fold increase at 4 weeks in 2 out of 5 from 9V, 14, 18C, 19F, and 23F	47 (18-76)	48	2 fold increase seen in 67% of patients. 4 fold increase in 47%	Poor response in MTX. Combined TNFi and MTX not worse than MTX monotherapy.
Mori 2012	MTX (62)<br>TOC + MTX (54)<br>TOC (50)<br>Control 24	23 valent	≥2-fold in IgG concentrations or ≥10- fold or more increase in opsonisation indices	MTX 68.3<br>TOC + MTX 65.1<br>TOC 68.3<br>Control 69.2	MTX 51 (82.3),<br>TOC + MTX (92.6)<br>TOC 43 (86)<br>Control (79.2)	Fold increases 6b/23f (SD) MTX 1.5 (1.1-3.0)/ 2.6 (1.4-4.1 TOC + MTX 1.6 (1.2-1.9)/ 2.9 (1.0-6.9) TOC 2.8 (1.4-4.4)/ 3.4 (1.5-6.8) Control 1.8 (1.3-3.7)/ 3.5 (1.7-5.6)	Post-vaccination Ab responses reduced when TOC combined with MTX.
Mori 2022	MTX(10)<br>MTX+JAK(23)<br>JAK (20)	PCV13	2 fold increase in IgG 4-6 weeks post vaccination	MTX 60.0 (2.4), JAKi 65.9(9.5), MTX+JAKi 68.6 (8.0)	MTX(90,), JAKi (80), MTX+JAKi (73.9)	GMFI fold increase: MTX 9.30(3.90-22.20), JAKi 6.36 (4.48-8.85), MTX+JAKi 3.46 (2.08-4.66)	MTX + JAKi has reduced antibody response (52.2%). JAK alone does not impair response (95%).
O'dell 1996	MTX (20)<br>Control (20)	23 valent	Ab titres >300ng/ml or ≥ 2-fold increase compared to prevaccine titres Type 3, 7F, 9N and 14 serotypes	-	60	Control response (77)<br>MTX (50)	MTX poor response versus control group

Racokzi 2016	ETA+MTX (15)<br>ETA (7)<br>HC (24)	PCV13	ARR ≥ 2, 4-weeks post-vaccination	RA 55.1 (10.4)<br>HC 63.9 (9.8)	ETA (77.3)<br>HC (75)	IgG levels 4 weeks post vaccination: ETA: 2.6-fold (±1.5) increase, HC 6.13-fold (± 7.3) increase.	Reduced response rate in TNFi exposed group compared to HC. No significant adverse effect of MTX + ETA combination compared to ETA monotherapy.
Tsuru 2014	Toculizumab (21)	23 valent	≥2-fold increase in Ab titres in at least 6 of 12 measured serotypes	54	81	100% patients achieved adequate Sero-response	No comparator group.
